# Dual Roles of Signal Transducer and Activator of Transcription 1 (STAT1) in Cancer: Expression Patterns, Prognostic Significance, and Immune Associations Across Multiple Tumor Types

**DOI:** 10.7759/cureus.92471

**Published:** 2025-09-16

**Authors:** Ebtihal Kamal, Samah O Mohager

**Affiliations:** 1 Department of Basic Medical Sciences, College of Medicine, Prince Sattam Bin Abdulaziz University, Al-Kharj, SAU; 2 Department of Basic Medical Sciences, Faculty of Medicine, Prince Sattam Bin Abdulaziz University, Al-Kharj, SAU

**Keywords:** bioinformatics, enrichment analyses, immune cell infiltration, protein expression, stat1

## Abstract

Background: Signal transducer and activator of transcription 1 (STAT1) is a pivotal transcription factor that plays a dual role in cancer biology. It behaves as a tumor suppressor and, under certain conditions, as a promoter. Few studies have analyzed STAT1 in pan-cancers and explored its role in cancer biology. This study aimed to investigate the expression of STAT1 across various cancers at different stages. Additionally, we examined the promoter methylation of STAT1 and its impact on STAT1 expression, evaluated the functional states of STAT1 in different cancer types, analyzed the correlation between STAT1 expression and survival prognosis, and studied the tumor immune infiltration. Furthermore, we analyzed STAT1 expression across diverse immune subsets in cancer. Subsequently, we conducted an analysis of STAT1-protein interactions and studied the cancer hallmarks of STAT1 and its related proteins.

Methods: Various bioinformatics tools (Gene Expression Profiling Interactive Analysis (GEPIA), Tumor Immune Estimation Resource (TIMER), University of Alabama at Birmingham Cancer Data Analysis Portal (UALCAN), Kaplan-Meier Plotter, StarBase, Enrichr, and Tumor and Immune System Interaction Database (TISIDB)) and databases (Cancer Single-cell State Atlas (CancerSEA) and Search Tool for the Retrieval of Interacting Genes/Proteins (STRING)) were used in this study. We comprehensively investigated STAT1 protein expression across various types of cancer and the correlation between its expression and different tumor stages, DNA methylation, survival outcomes, and immune cell infiltration. Additionally, we examined the correlation between STAT1 expression and various immune subtypes of cancer, as well as the functional states of STAT1 in different cancer types. Moreover, we investigated the STAT1 protein interactions and cancer hallmarks of STAT1 and its related proteins.

Results: High STAT1 expression was found in 21 cancers and was differentially expressed across different clinical tumor stages and promoter methylation levels. In addition, we found that high expression correlated with better overall survival in some tumors, whereas it correlated with worse overall survival in other tumor types. Furthermore, the expression of STAT1 was associated with the level of immune infiltration in multiple tumors and different immune subtypes. We found that STAT1 expression had dual correlations (positive and negative) with different functional states in tumors, including angiogenesis and apoptosis. STAT1 protein interaction and Kyoto Encyclopedia of Genes and Genomes (KEGG) enrichment analyses revealed that 10 STAT1-interacting proteins were significantly enriched in cancer-related pathways.

Conclusion: Our findings suggest that STAT1 may serve as a potential diagnostic and prognostic marker for several cancer types.

## Introduction

Signal transducer and activator of transcription 1 (STAT1) is a pivotal protein that plays a crucial role in various cellular mechanisms, including the immune response [1] and tumorigenesis [2,3]. It is primarily activated by interferons, which are crucial for immune responses [4], and regulates the genes involved in apoptosis, cell cycle arrest, and immune modulation [5].

Its role in cancer is complex, as it can act as a tumor promoter or suppressor, depending on the type of cancer [5]. STAT1 may contribute to tumor progression by regulating oncogenic pathways involving MYC, which drives cancer cell proliferation [6]. Additionally, elevated STAT1 levels are associated with increased expression of pro-inflammatory cytokines, which further supports tumor growth and metastasis [7]. In sarcoma, STAT1 acts as a tumor promoter by suppressing the expression of the pro-apoptotic factors (Fas and Bad), thereby increasing resistance to apoptosis. This mechanism facilitates sarcoma development by allowing tumor cells to evade programmed cell death, a crucial process for maintaining tumor growth and progression [8]. However, in certain contexts, STAT1 suppresses tumors by enhancing the immune response against tumors by promoting cytotoxic T lymphocyte (CTL) activity [9], inhibiting angiogenesis [10], and controlling tumor growth by triggering apoptosis in tumor cells through the activation of various pro-apoptotic genes, such as p53, Bax, Fas, Noxa [11], and tumor necrosis factor-related apoptosis-inducing ligand (TRAIL) [12].

Evidence supporting the role of STAT1 as a suppressor of breast cancer is provided by the observation that STAT1 expression is diminished in tumor epithelial cells compared to normal breast tissue [13]. Therefore, understanding the specific context of STAT1 expression and its downstream effects in each cancer is crucial for developing effective treatment protocols, particularly in immunotherapy and chemotherapy.

Recent studies have highlighted the significance of STAT1 in multiple cancers. The expression of STAT1 is a diagnostically valuable biomarker for various malignancies. Notably, elevated levels of STAT1 mRNA correlate with enhanced tumor invasiveness and an unfavorable prognosis in bladder cancer [14], kidney renal papillary cell carcinoma (KIRP) [15], gliomas [16], and stomach adenocarcinoma (STAD) [17]. Conversely, high STAT1 levels are correlated with better overall survival (OS) in ovarian cancer [18].

Although extensive research has focused on the role of STAT1 in individual cancers, there have been limited reports on the role of STAT1 in pan-cancer analysis [19]. Therefore, it is important to investigate the role of STAT1 in different tumor types using pan-cancer analysis. While STAT1 has been implicated in various aspects of cancer biology, significant gaps persist in understanding its complex roles, particularly concerning its dual functionality, interactions with other pathways, and potential as a therapeutic target. Further research in these areas could enhance the understanding of cancer mechanisms and improve treatment outcomes.

In the present study, we comprehensively utilized various databases to study STAT1 expression in different tumors at various stages. Epigenetic mechanisms, which are both heritable and reversible, encompass alterations in DNA methylation, histone modifications, and small non-coding microRNAs (miRNAs) [20]. Methylation of gene promoters is a significant epigenetic modification that can lead to the silencing of genes, particularly tumor-suppressor genes. Methylation of gene promoters plays a crucial role in cancer development [20]. In the current study, we compared methylation data with the gene expression of STAT1 and investigated the relationship between the methylation level of STAT1 and different types of tumors. Next, we analyzed the correlation between STAT1 expression and survival prognosis, as well as tumor immune infiltration. Moreover, we used a variety of bioinformatics tools to study STAT1 expression in different immune subsets of cancer and the functional states of STAT1 in various cancers.

Subsequently, we analyzed STAT1 protein interactions and functional enrichment. By investigating these co-expressed genes and pathways, a more comprehensive understanding of their biological functions can be achieved.

## Materials and methods

**Figure 1:**
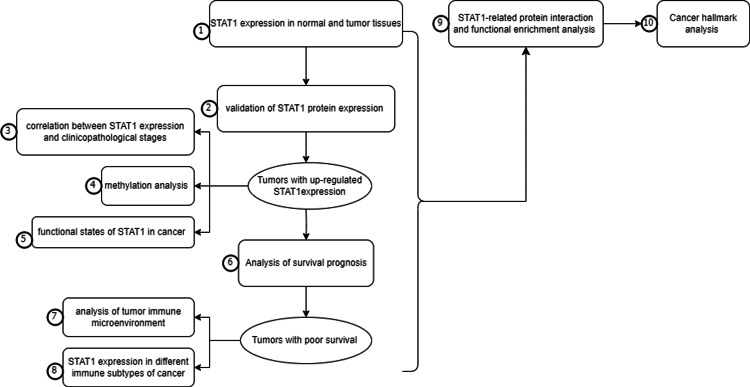
Workflow of the study.

All the steps in this study were summarized in Figure 1

Gene expression in normal tissues and cancer

GeneCards is a comprehensive web-based database of human genes. It contains genomic, proteomic, and transcriptomic data for 73,000 human genes [21]. In our study, we obtained STAT1 protein expression information in normal human tissues from the GTEx, BioGPS, and SAGE databases according to the GeneCards website (www.genecards.org).

The Gene Expression Profiling Interactive Analysis (GEPIA) is a web-based tool that provides customizable analysis of gene expression data from cancer and normal tissues, primarily using datasets from Cancer Genome Atlas (TCGA) and GTEx [22]. We used GEPIA to explore the expression of STAT1 in tumor and normal tissues. Statistical significance was set at p < 0.05.

Validation of protein levels in the HPA database

The Human Protein Atlas (HPA) is available online. It maps all human proteins in cells, tissues, and organs by integrating various omics-based technologies. Additionally, it employs various histological techniques, including antibody-based imaging, mass spectrometry-based proteomics, and transcriptomics [23]. We used HPA to validate the differences in STAT1 expression at the protein level. We downloaded and compared the immunohistochemistry (IHC) images of STAT1 protein in normal and tumor tissues with upregulated STAT1 expression. STAT1 was stained with antibodies HPA000931, HPA000982, and CAB004049. Staining intensity (negative, weak, moderate, or strong) and the fraction of stained cells (<25%, 25-75%, or >75%) were evaluated using the HPA website to assess differences in STAT1 expression at the protein level.

Correlations between STAT1 expression and clinicopathological stage

The University of Alabama at Birmingham Cancer Data Analysis Portal (UALCAN) database is a highly accessible and robust tool. This database is an OMICS tool specifically designed to analyze the transcriptome of cancer. It includes RNA-seq expression data obtained from the TCGA dataset [24]. UALCAN was used to ascertain the correlation between gene expression and clinicopathological stage. We used the “expression” module of UALCAN to investigate the profile of STAT1 expression at different tumor stages.

Methylation analysis

Methylation analysis is an essential technique for investigating the regulation of gene expression in cancer. We investigated the TCGA module in the UALCAN database to understand the relationship between STAT1 gene expression and DNA methylation. We aimed to explore the methylation levels of the STAT1 promoter in different types of cancers and analyze how methylation patterns change as cancer progresses through different stages.

Functional states of STAT1 in various cancer types 

Cancer Single-cell State Atlas (CancerSEA) is a dedicated database that aims to comprehensively decode the distinct functional states of cancer cells at single-cell resolution [25]. We used CancerSEA to study the functional status of STAT1 in various cancers. The correlation between the functional states in cancers with upregulated STAT1 was explored, including invasion, metastasis, proliferation, epithelial-mesenchymal transition (EMT), angiogenesis, apoptosis, cell cycle, differentiation, DNA damage, DNA repair, hypoxia, inflammation, quiescence, and stemness. We set the threshold for the correlation between STAT1 and cancer functional states at a correlation strength of 0.3 and a p-value of less than 0.05.

Analysis of survival prognosis

The Kaplan-Meier plotter is an online tool designed to analyze the impact of gene expression on survival outcomes in various cancers. It was used to generate survival curves based on the GEO, EGA, and TCGA databases. It compares the difference in survival rate between the high- and low-expression groups [26]. We used the Kaplan-Meier plotter to analyze the effect of STAT1 expression on OS in different types of cancers by calculating the 95% confidence interval and p-value.

StarBase is a comprehensive biological resource designed to facilitate the exploration of RNA interactions, focusing on miRNA and long non-coding RNA (lncRNA) interactions with messenger RNAs (mRNAs). It integrates data from various high-throughput sequencing techniques, such as CLIP-Seq and Degradome-Seq, to provide detailed interaction maps that help in understanding the regulatory roles of these non-coding RNAs in gene expression and cancer biology [27]. We used StarBase to perform analyses that elucidated the regulatory mechanisms underlying gene expression and their implications for patient prognosis in skin cutaneous melanoma (SKCM) and low-grade glioma (LGG), which were not found in the Kaplan-Meier plotter.

Analysis of the tumor immune microenvironment

Tumor Immune Estimation Resource (TIMER) is an integrated web-based tool designed to analyze tumor-infiltrating immune cells in various cancer types [28]. It provides dynamic analysis and visualization of gene expression data. We used the TIMER algorithm to explore the correlation between STAT1 gene expression in tumors and immune infiltration, as determined by Spearman’s test, and created a scatter diagram. Statistical significance was set at p < 0.05.

STAT1 expression in different immune subtypes of cancers 

The Tumor and Immune System Interaction Database (TISIDB) integrates various data types to assess the interactions between cancer and the immune system [29]. We studied the associations between STAT1 expression and immune subtypes in cancers with upregulated expression of STAT1 protein using the “subtype” module of the TISIDB database. STAT1 mRNA expression was investigated in different immune subtypes, including C1 (wound healing), C2 (IFN-γ dominant), C3 (inflammatory), C4 (lymphocyte-depleted), C5 (immunologically quiet), and C6 (TGF-β dominant).

STAT1-related protein interactions and functional enrichment analysis

Search Tool for the Retrieval of Interacting Genes/Proteins (STRING) online tool is a comprehensive resource that integrates known and predicted protein-protein interactions, both direct (physical) and indirect (functional) associations [30]. We utilized the STRING database to visualize the STAT1 protein interaction using the protein-by-name module of STRING; the input was the STAT1 protein, and the output was a graphical representation of protein interactions. A confidence score > 0.7 was set as the significance threshold, and the maximum number of interactors was set to 50. Additionally, we used STRING to identify the enriched Kyoto Encyclopedia of Genes and Genomes (KEGG) pathways associated with STAT1-related proteins. Next, we imported the protein-protein interaction (PPI) network into Cytoscape (version 3.8.2) for visualization and analysis of the interactions. The cytoHubba plugin within Cytoscape was used to identify key modules, and the top 10 nodes ranked by the Maximum Clique Centrality (MCC) method from cytoHubba were considered hub genes.

Enrichr is a powerful web-based tool designed for gene set enrichment analysis. Enrichr contains an extensive collection of annotated gene sets, systematically organized into libraries. These libraries span a diverse range of categories, including pathways, diseases, cell types, and gene ontology (GO) terms [31]. We conducted GO function enrichment analyses for the 10 hub genes that closely interact with STAT1, which were obtained from the cytoHubba plugin within Cytoscape, using the Appyters feature of the Enrichr platform for enhanced visualization and analysis.

Cancer hallmark analysis

CancerHallmarks.com established a consensus list of cancer hallmark genes by merging 6,763 genes from the available mapping resources. This enhances the utility of the hallmark concept as an effective organizational tool by funneling genes/proteins into biological functions [32]. We explored the potential hallmarks of cancer associated with STAT1-related proteins using the Cancer Hallmarks website. A radial plot was constructed to illustrate the enrichment of key oncogenic hallmarks associated with STAT1-related proteins. In this plot, each spoke represents a distinct oncogenic hallmark, including tumor-promoting inflammation, sustaining proliferative signaling, evading immune destruction, resisting cell death, evading growth suppressors, and genome instability. The first layer of data represents the enrichment of cancer hallmarks compared to the reference set of genes. In this overrepresentation analysis, differently colored slices represent each of the 10 cancer hallmarks, and only the significant ones (adjusted p < 0.05) are colored. The radial distance from the center to the tip of each spoke indicates the adjusted p-value, with longer distances indicating greater statistical significance. The adjusted p-values are displayed on a logarithmic scale to enhance interpretability.

## Results

Analysis of STAT1 expression in tumor and normal tissues 

**Figure 2:**
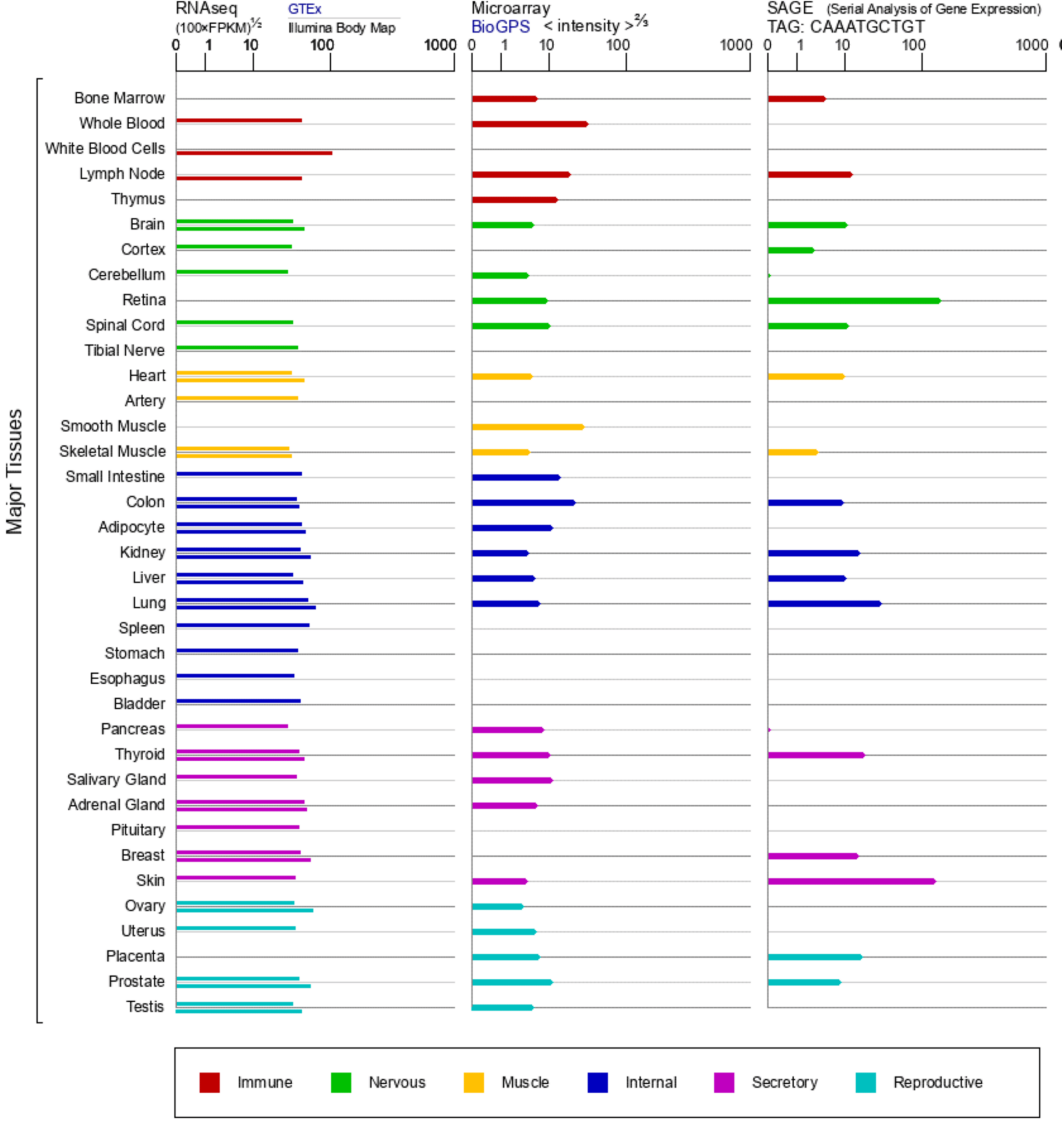
STAT1 mRNA expression in normal human tissues in the GTEx, Illumina, BioGPS, and SAGE databases. STAT1: Signal transducer and activator of transcription 1

The expression of STAT1 protein in human tissues was analyzed using the GTEx, BioGPS, and SAGE databases from the GeneCards website. According to the GTEx database, STAT1 protein was expressed differently in different tissues, with the highest expression in white blood cells, and according to the BioGPS database, in smooth muscle. In the SAGE databases, the retina and skin are the tissues with the highest STAT1 mRNA expression in the human body (Figure 2).

**Figure 3:**
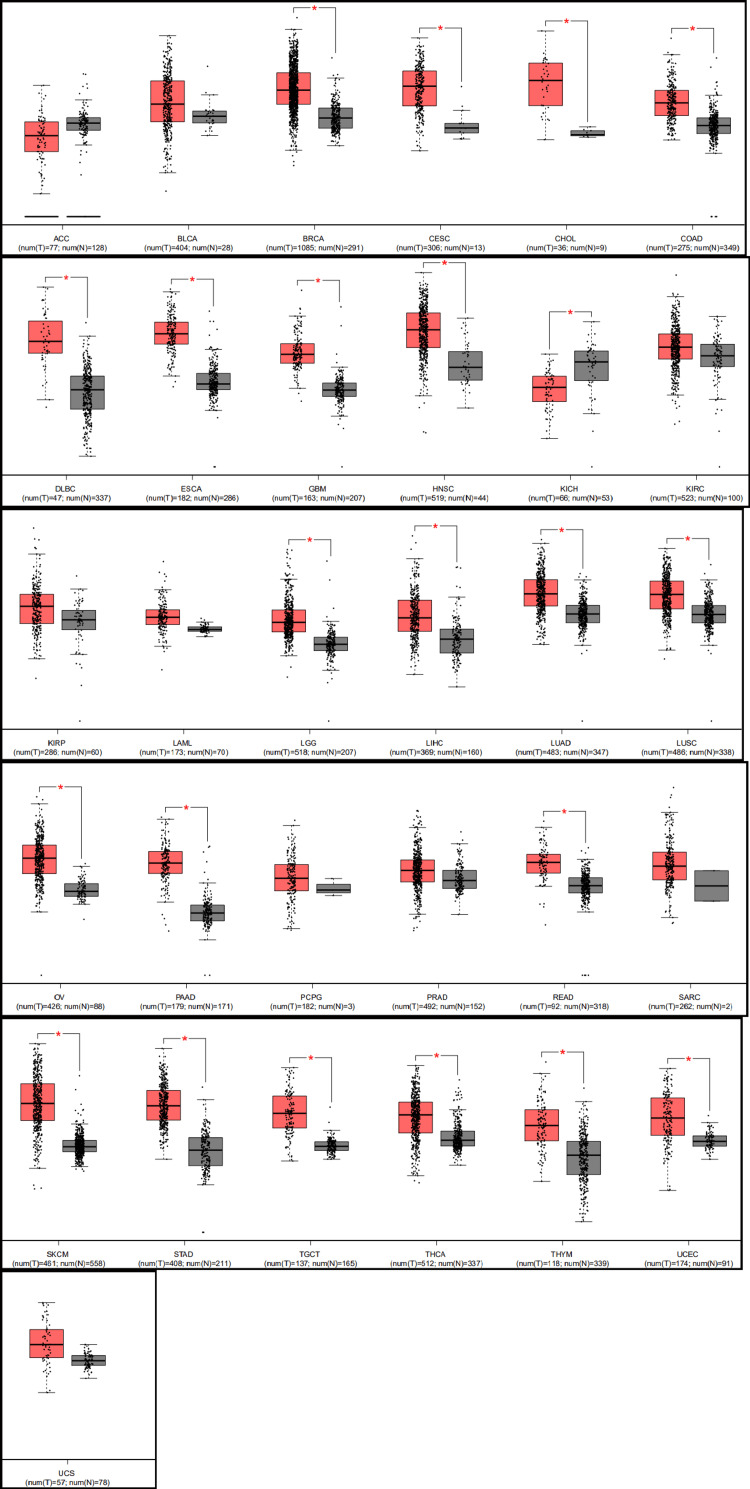
STAT1 expression profile across different cancer types using the GEPIA database. Distributions of STAT1 expression levels are displayed using box plots; red box plots are for tumors, and grey box plots indicate normal tissues. * p-value < 0.05 HPA: Human Protein Atlas; STAT1: Signal transducer and activator of transcription 1; BRCA: breast invasive carcinoma; CESC: cervical squamous cell carcinoma; ESCA: esophageal carcinoma; HNSC: head and neck squamous cell carcinoma; LUAD: lung adenocarcinoma; STAD: stomach adenocarcinoma; UCEC: uterine corpus endometrial carcinoma; LGG: low-grade gliomas; LIHC: liver hepatocellular carcinoma; PAAD: pancreatic adenocarcinoma; READ: rectum adenocarcinoma; OV: ovarian cancer; KICH: kidney chromophobe

Our analysis using GEPIA revealed that the expression of STAT1 was significantly upregulated in 21 cancers. The significant cancers were breast invasive carcinoma (BRCA), CESC, cholangiocarcinoma (CHOL), colonic adenocarcinoma (COAD), diffuse large B-cell lymphoma (DLBC), ESCA, glioblastoma multiforme (GBM), head and neck cancer (HNSC), LGG, liver hepatocellular carcinoma (LIHC), LUAD, lung squamous cell carcinoma (LUSC), ovarian cancer (OV), PAAD, rectum adenocarcinoma (READ), SKCM, STAD, testicular germ cell tumor (TGCT), thyroid cancer (THCA), thymoma (THYM), and UCEC compared with normal tissues. However, STAT1 expression was significantly downregulated in kidney chromophobe (KICH) tissues compared to that in normal kidney tissues (Figure 3).

**Figure 4:**
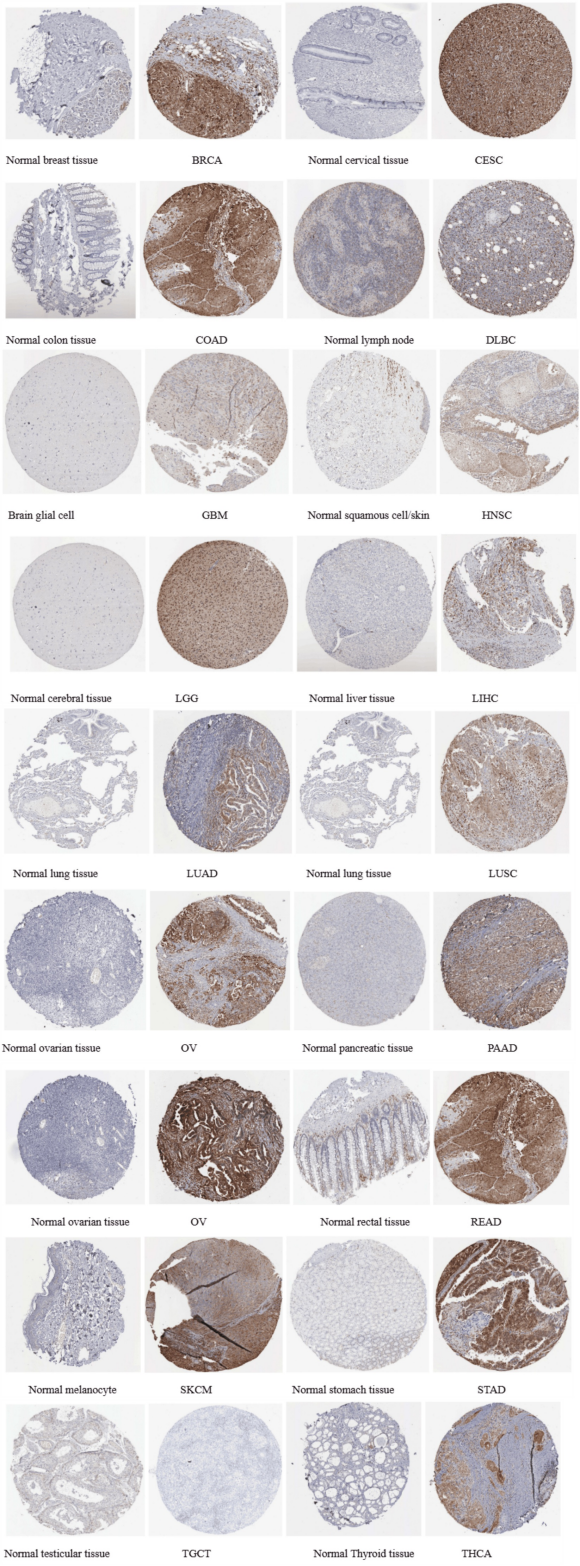
Levels of STAT1 in different tumors. The results were based on the HPA database. HPA: Human Protein Atlas; STAT1: Signal transducer and activator of transcription 1; BRCA: breast invasive carcinoma; CESC: cervical squamous cell carcinoma; ESCA: esophageal carcinoma; HNSC: head and neck squamous cell carcinoma; LUAD: lung adenocarcinoma; STAD: stomach adenocarcinoma; UCEC: uterine corpus endometrial carcinoma; LGG: low-grade gliomas; LIHC: liver hepatocellular carcinoma; PAAD: pancreatic adenocarcinoma; READ: rectum adenocarcinoma; OV: ovarian cancer; KICH: kidney chromophobe

The protein expression level of STAT1 was verified using the HPA database (Figure 4).

Results of the correlation analysis of STAT1 expression and pathological stages of tumors

**Figure 5:**
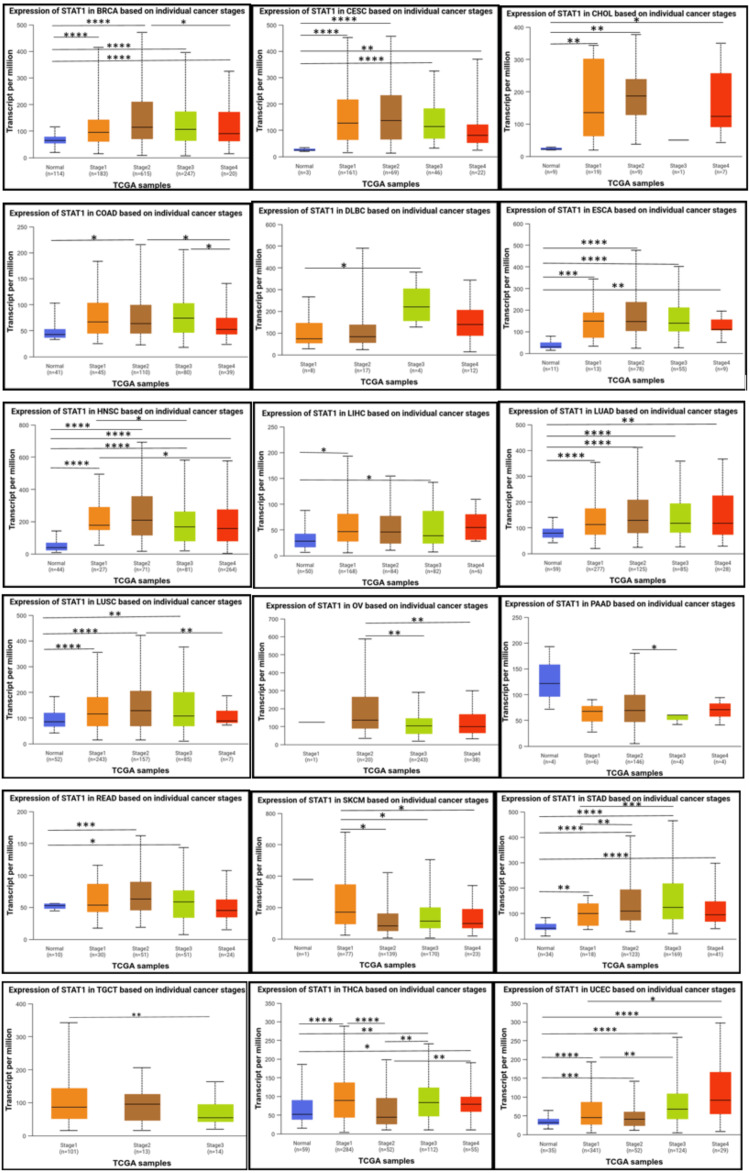
Correlation of STAT1 expression and pathological stages of tumors using the UALCAN database. Statistical significance is indicated by the number of stars (*: p < 0.05; **: p < 0.01; ***: p < 0.001). STAT1: Signal transducer and activator of transcription 1; UALCAN: University of Alabama at Birmingham Cancer Data Analysis Portal

According to the individual cancer stages, STAT1 was significantly upregulated in stages 1, 2, 3, and 4 compared to normal tissues in patients with BRCA, CESC, ESCA, HNSC, LUAD, STAD, and UCEC. Moreover, the expression of STAT1 was not significantly different between tumor stages and normal tissues in PAAD and SKCM. Despite the absence of a significant difference between normal tissue and various tumor stages in PAAD, a significant difference exists between stage 2 and stage 3. In SKCM, there is a significant difference between stage 1 and the other stages (2, 3, and 4)(Figure 5).

Methylation analysis

**Figure 6:**
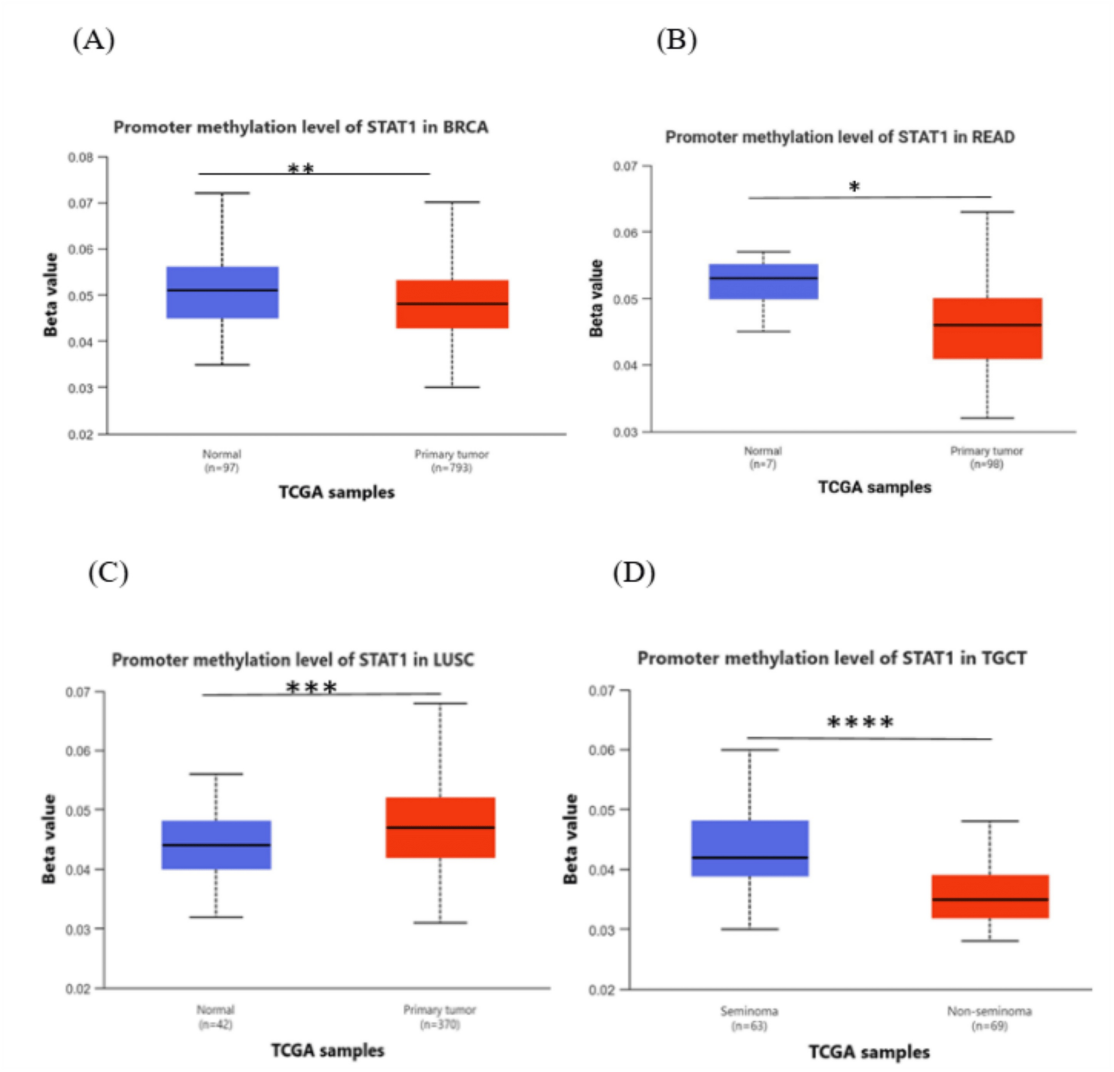
Promoter DNA methylation of STAT1 in normal and tumor samples of BRCA (A), and READ (B), LUSC (C), and TGCT (D). The UALCAN database was used. Statistical significance is denoted by the number of stars (*: p-value < 0.05; **: p-value < 0.01; ***: p-value < 0.001). BRCA: breast invasive carcinoma; LUSC: lung squamous cell carcinoma; READ: rectum adenocarcinoma; TGCT: testicular germ cell tumor; STAT1: Signal transducer and activator of transcription 1; UALCAN: University of Alabama at Birmingham Cancer Data Analysis Portal

The results from the UALCAN database, comparing cancer with normal tissues, showed that methylation levels of the STAT1 promoter in BRCA, READ, and TGCT were significantly lower than those in normal tissues (p < 0.05), and there was a negative correlation between STAT1 promoter methylation levels and STAT1 expression. In LUSC, the promoter methylation was significantly higher in the tumor in comparison to the normal tissue (Figure 6). In contrast, other cancer types yielded insignificant results (Appendix, Figure 17).

**Figure 7:**
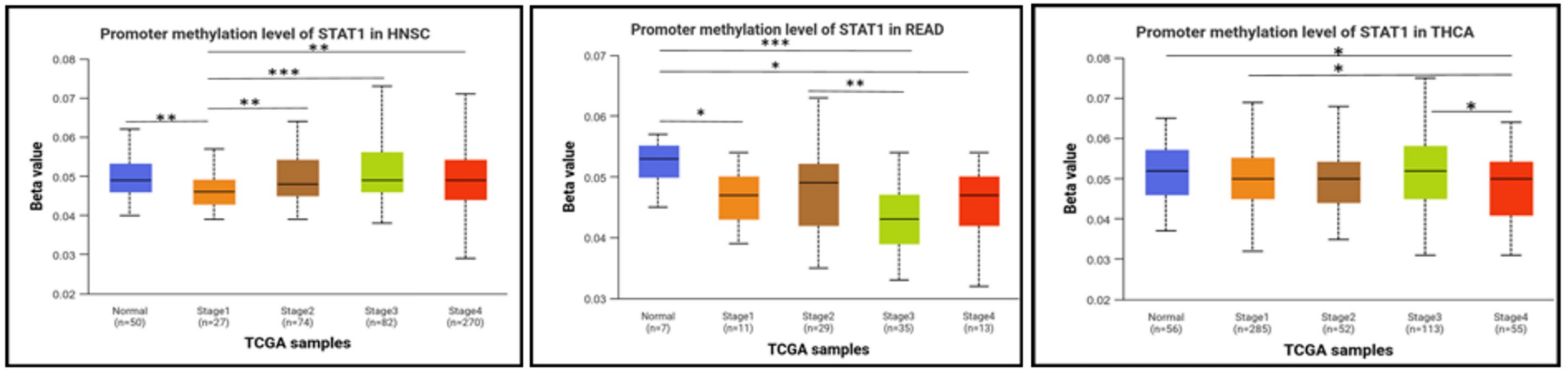
Promoter DNA methylation of STAT1 in normal and tumor samples based on individual cancer stages. Statistical significance is annotated by the number of stars (*: p-value < 0.05; **: p-value < 0.01; ***: p-value < 0.001). STAT1: Signal transducer and activator of transcription 1; HNSC: head and neck squamous cell carcinoma; READ: rectum adenocarcinoma; THCA: thyroid cancer; TCGA: Cancer Genome Atlas

Next, we studied the methylation patterns at different tumor stages. In HNSC, the promoter methylation levels were significantly different between normal and stages 1 and stage 1 and stages 2, 3, and 4. In addition, there were significant differences between stage 1 and stages 2, 3, and 4. In READ, there were significant differences between normal and stages 1, 3, and 4, and a significant difference between stages 2 and 3. In THCA, the promoter methylation of STAT1 was significantly different between normal and stage 4, and there was also a significant difference between stages 1 and 4 and stages 3 and 4 (Figure 7). The p-value of STAT1-promoter methylation in LUAD, PAAD, and SKCM was statistically insignificant between normal and all stages of tumors (Appendix, Figure 18).

Results of the functional states of STAT1

**Figure 8:**
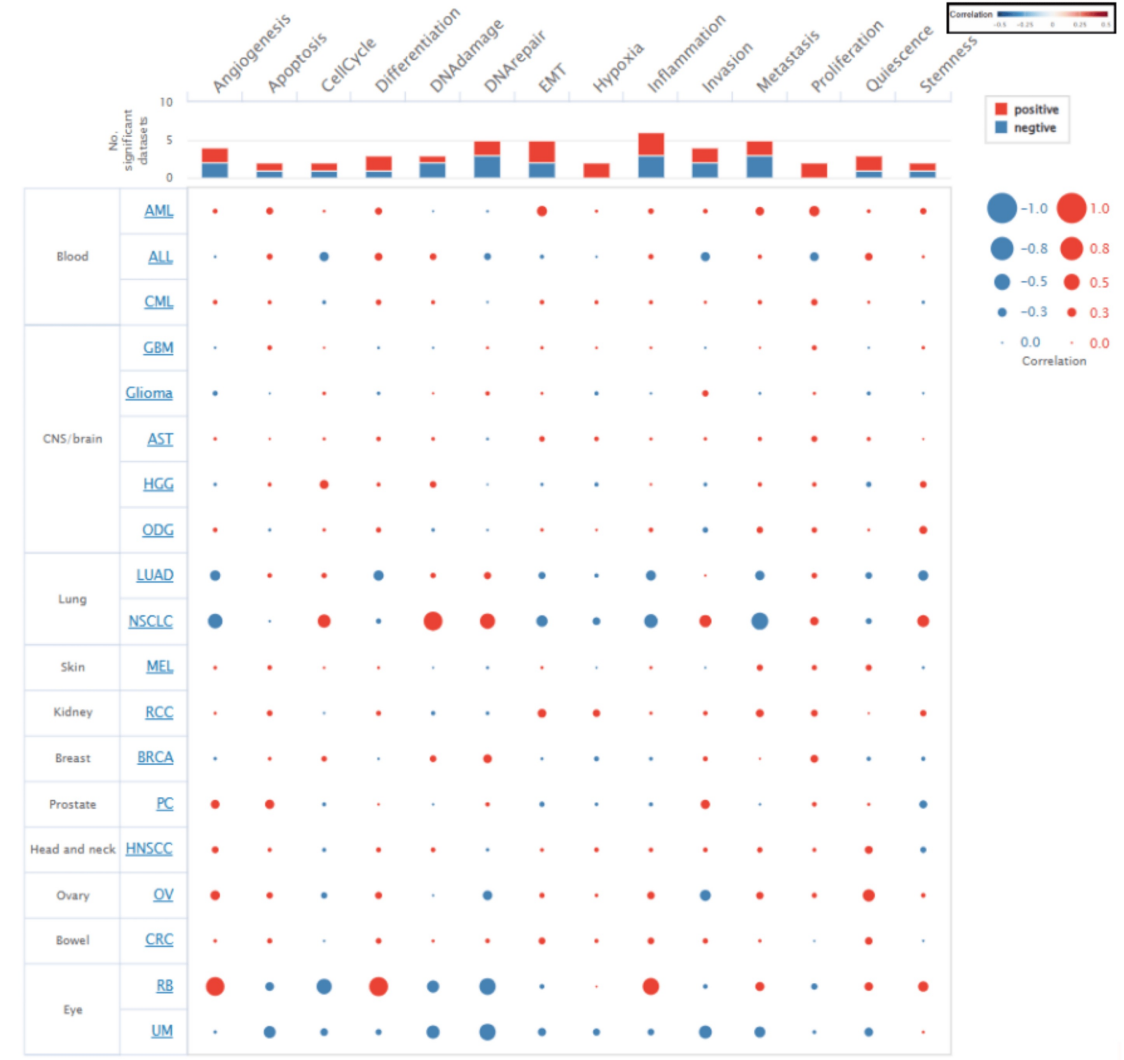
A bubble chart represents the correlation between STAT1 expression and functional states in cancer using CancerSEA. STAT1: Signal transducer and activator of transcription 1; CancerSEA: Cancer Single-cell State Atlas; BRCA: breast invasive carcinoma; CESC: cervical squamous cell carcinoma; ESCA: esophageal carcinoma; HNSC: head and neck squamous cell carcinoma; LUAD: lung adenocarcinoma; STAD: stomach adenocarcinoma; UCEC: uterine corpus endometrial carcinoma; LGG: low-grade gliomas; LIHC: liver hepatocellular carcinoma; PAAD: pancreatic adenocarcinoma; READ: rectum adenocarcinoma; OV: ovarian cancer; KICH: kidney chromophobe; TGCT: testicular germ cell tumor; THCA: thyroid cancer

We explored the functional state of STAT1 in different cancer types using CancerSEA. The correlation between STAT1 expression and functional states in various cancers is shown in Figure 8. Our research indicates that the expression of STAT1 can exhibit a positive correlation with a functional state in certain cancer types while demonstrating a negative correlation in others. Specifically, STAT1 expression is positively associated with angiogenesis and differentiation in retinoblastoma (RB). Conversely, it is linked to reduced angiogenesis and differentiation in non-squamous cell lung cancer (NSCLC) and LUAD.

Analysis of STAT1 gene expression profiles and their correlation with survival prognosis

**Figure 9:**
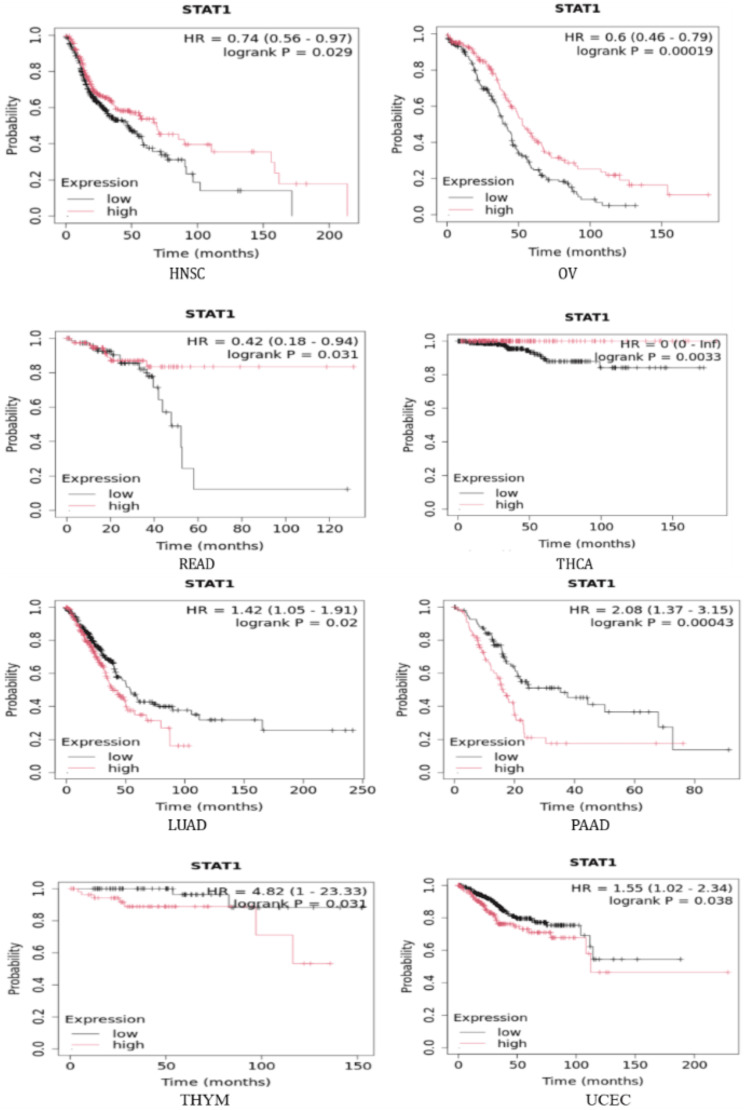
Correlation between STAT1 levels and patient survival outcomes. The Kaplan-Meier plotter for head and neck cancer (HNSC), ovarian cancer (OV), rectum adenocarcinoma (READ), thyroid cancer (THCA), lung adenocarcinoma (LUAD), pancreatic adenocarcinoma (PAAD), thymoma (THYM), and uterine corpus endometrial carcinoma (UCEC) STAT1: Signal transducer and activator of transcription 1

**Figure 10:**
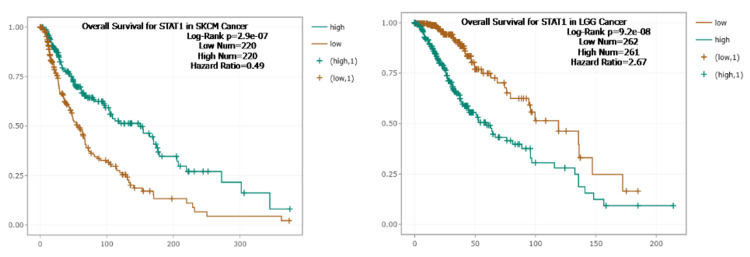
Correlation between STAT1 levels and patient survival outcomes. StarBase database for skin cutaneous melanoma (SKCM) and low-grade glioma (LGG). STAT1: Signal transducer and activator of transcription 1

To further investigate the correlation between STAT1 expression and tumor prognosis, we used the Kaplan-Meier plotter and StarBase database to study the correlation between STAT1 and OS in tumors with high expression of STAT1 protein (BRCA, CESC, CHOL, COAD, DLBC, ESCA, GBM, HNSC, LGG, LIHC, LUAD, LUSC, OV, PAAD, READ, SKCM, STAD, TGCT, THCA, THYM, and UCEC) (Appendix, Figure 19). We found that upregulation of STAT1 in HNSC, OV, READ, and THCA in Kaplan-Meier plotter (Figure 9) and SKCM in StarBase database (Figure 10) was associated with better OS. While upregulation of STAT1 in LUAD, PAAD, THYM, and UCEC in Kaplan-Meier plotter (Figure 9) and LGG in StarBase database (Figure 10) was associated with poor prognosis and worse OS (Figure 9). In this study, we further analyzed the tumor with high expression of STAT1 protein, and its expression is associated with poor prognosis, namely, LGG, LUAD, PAAD, THYM, and UCEC.

Immune cell infiltration of STAT1 in cancers

**Figure 11:**
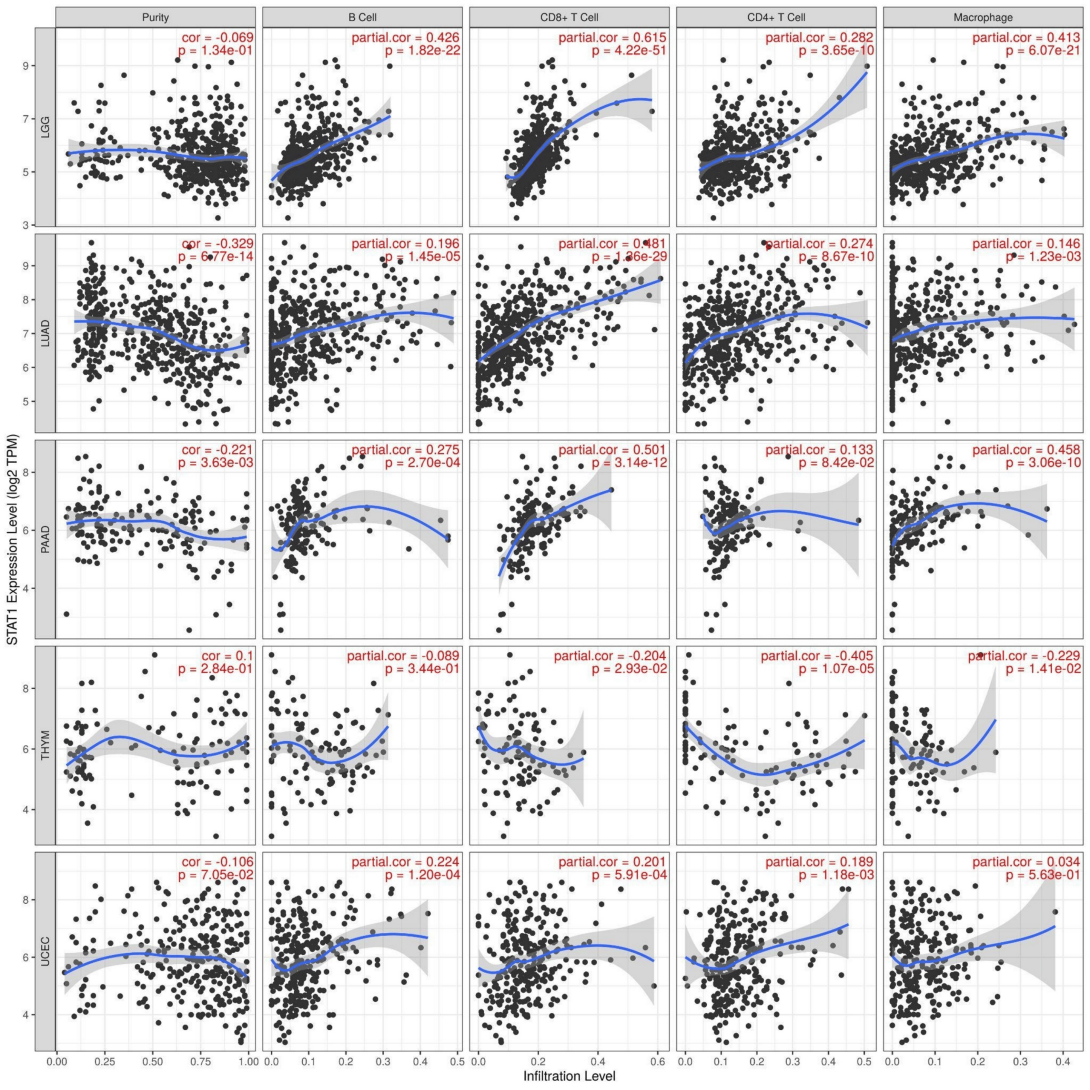
Correlation between STAT1 expression and immune infiltration levels in tumors with upregulated STAT1 expression associated with worse prognosis using the TIMER database. STAT1: Signal transducer and activator of transcription 1; TIMER: Tumor Immune Estimation Resource

We used the TIMER database to study the correlation between STAT1 and immune cell infiltration in tumors with upregulated STAT1 expression and associated with worse prognosis (LGG, LUAD, PAAD, THYM, and UCEC). The results showed that the expression of STAT1 has a significant positive correlation with the infiltration of B cells, CD8+ cells, CD4+ cells, and macrophages in LGG, LUAD, PAAD, THYM, and UCEC. In particular, the expression of STAT1 demonstrated a significant positive correlation with the infiltration of B cells, CD8+ cells, CD4+ cells, and macrophages in LGG, LUAD, and PAAD. Furthermore, we identified a significant positive correlation between STAT1 expression and the infiltration of B cells, CD8+ cells, and CD4+ cells in UCEC. Conversely, in THYM, the expression of STAT1 was significantly negatively correlated with the infiltration of CD8+ cells, CD4+ cells, and macrophages (Figure 11).

STAT1 expression in different immune subtypes

**Figure 12:**
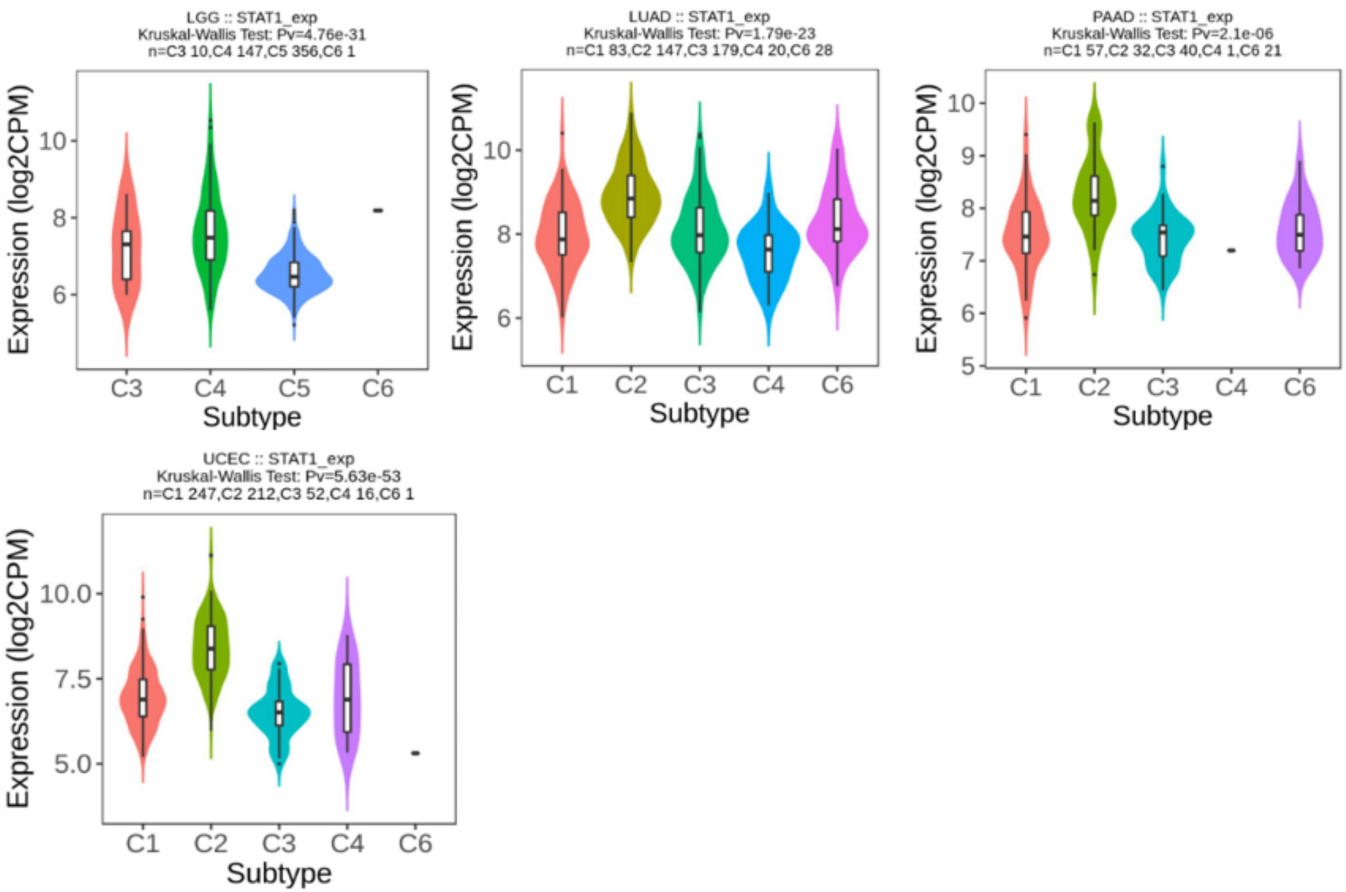
Correlation between STAT1 expression and immune subtypes in tumors with upregulated STAT1 expression, associated with a worse prognosis. C1 (wound healing), C2 (IFN-γ-dominant), C3 (inflammatory), C4 (lymphocyte-depleted), C5 (immunologically quiet), and C6 (TGF-β-dominant) STAT1: Signal transducer and activator of transcription 1; lung adenocarcinoma (LUAD), pancreatic adenocarcinoma (PAAD), and uterine corpus endometrial carcinoma (UCEC)

We used the TISIDB database to analyze STAT1 expression in the immune subtypes of tumors with upregulated STAT1 expression, which was associated with a worse prognosis. The results show that STAT1 expression differed significantly in four of the five tumors for immune subtypes, LGG (four subtypes), LUAD (five subtypes), PAAD (five subtypes), and UCEC (five subtypes), as presented in Figure 12.

The PPI and functional enrichment of STAT1 in cancers

**Figure 13:**
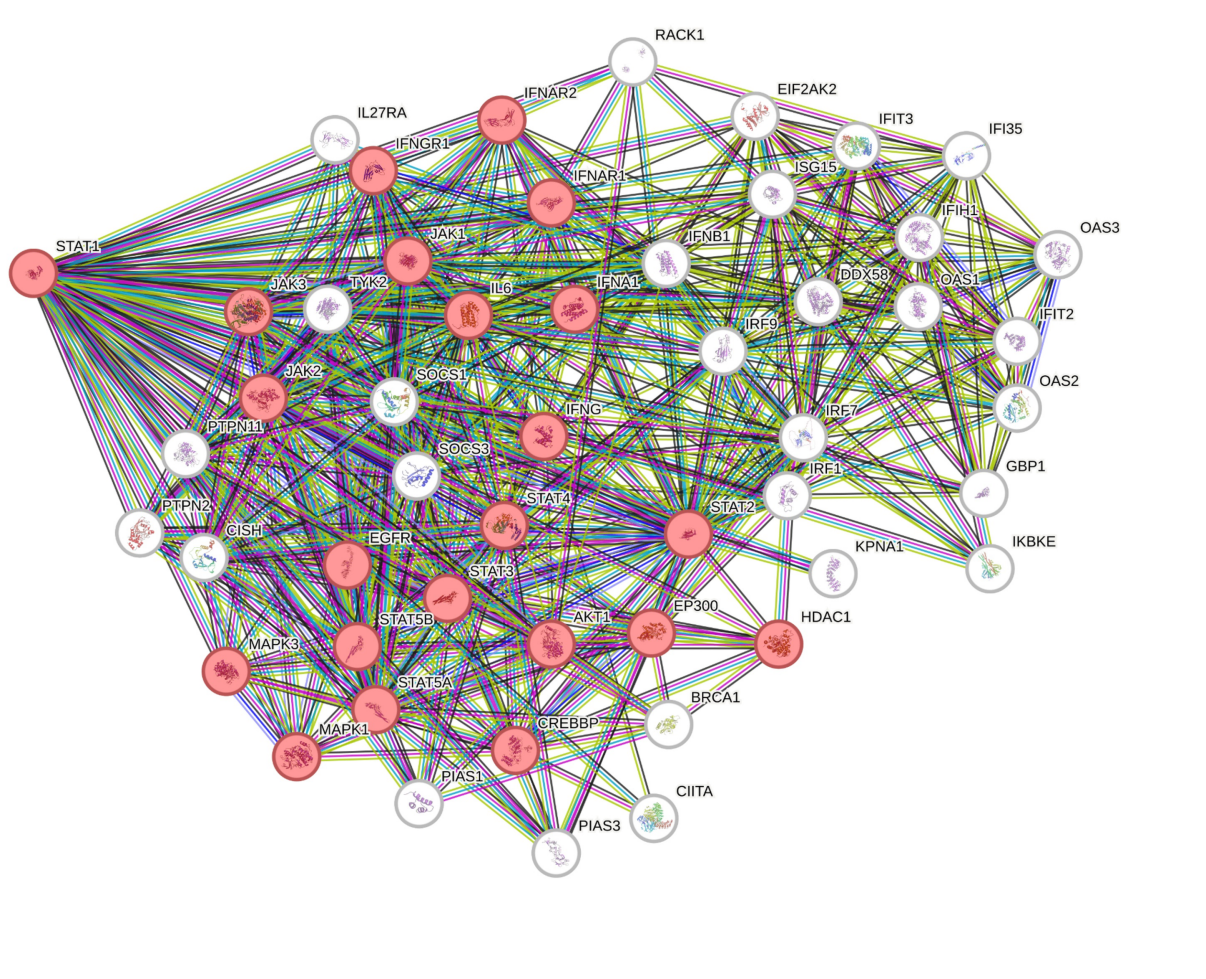
STAT1 protein interaction, using the STRING database. STRING: Search Tool for the Retrieval of Interacting Genes/Proteins

**Figure 14:**
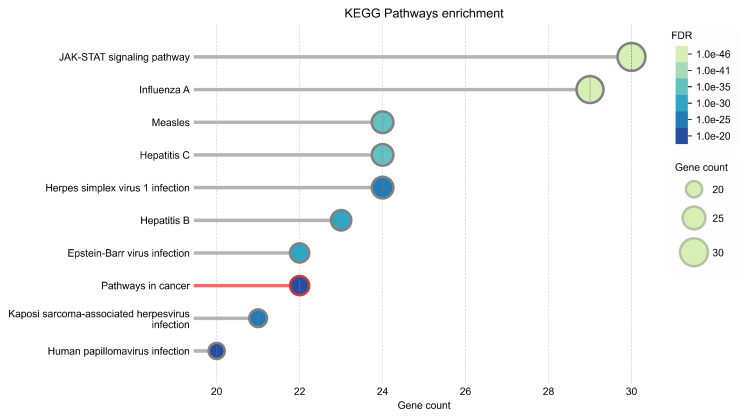
KEGG pathway analysis of the top 50 proteins interacting with STAT1 using the STRING database. KEGG: Kyoto Encyclopedia of Genes and Genomes; STAT1: Signal transducer and activator of transcription 1; STRING: Search Tool for the Retrieval of Interacting Genes/Proteins

The PPI network was constructed using STRING (Figure 13). The top 50 proteins interacting with STAT1 are shown in Figure 12. KEGG enrichment analysis was performed, and the top 50 interacting proteins were screened according to the p-value, from the smallest to the largest (Figure 14).

**Figure 15:**
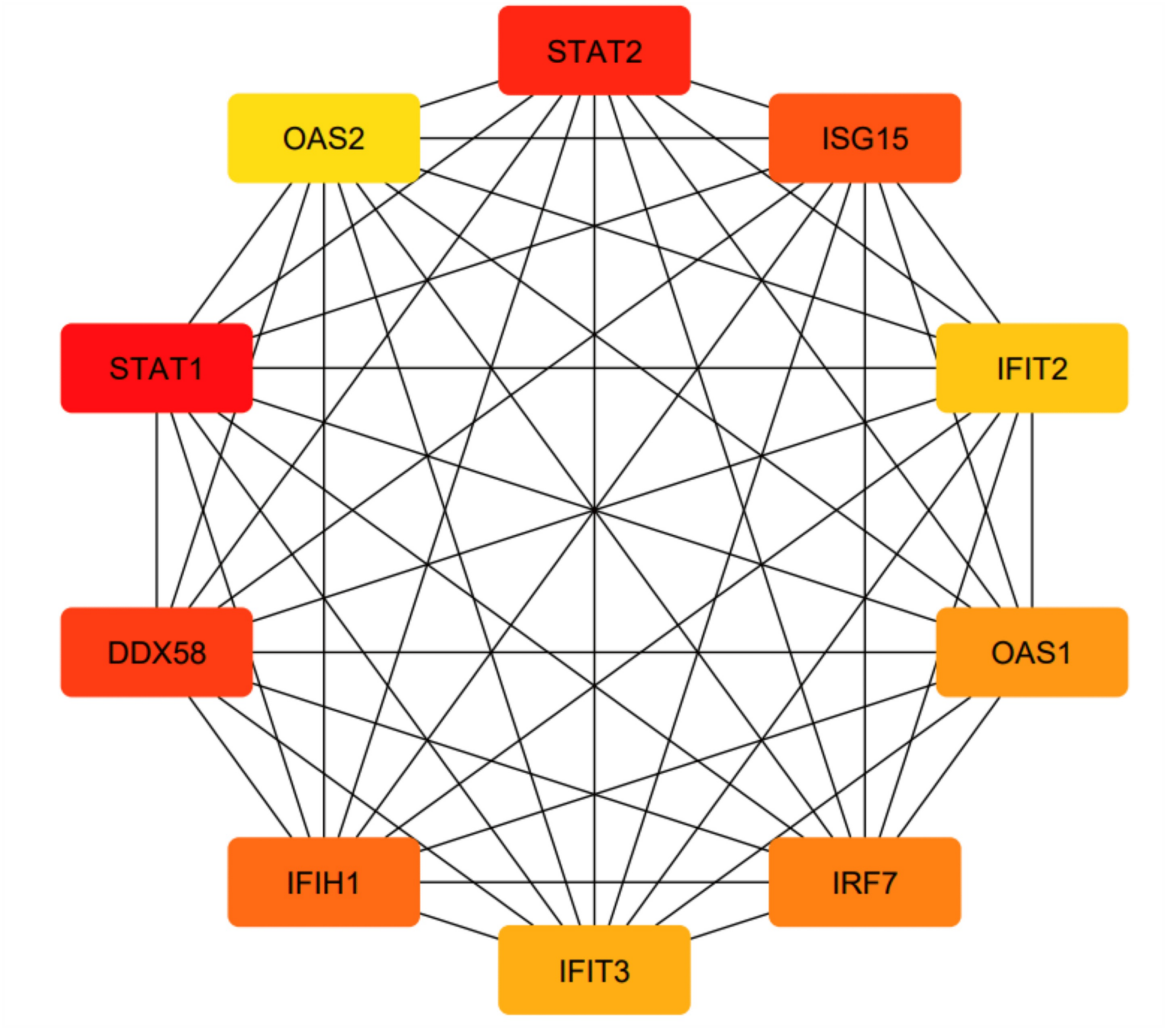
Top ten hub genes of the STAT1 PPI network. STAT1: Signal transducer and activator of transcription 1; PPI: protein-protein interaction

Moreover, we constructed a PPI network using Cytoscape (Appendix, Figure 20) and identified 10 hub genes using the cytoHubba plug-in (Figure 15).

Next, we performed GO enrichment analysis of the 10 hub genes using the Appyters feature of Enrichr. The results showed that the 10 hub genes were significantly enriched in the following biological processes: defense response to virus, positive regulation of type I interferon production, positive regulation of interferon-beta production, regulation of interferon-beta production, antiviral innate immune response, negative regulation of viral processes, interleukin-27-mediated signaling pathway, type I interferon-mediated signaling pathway, cellular response to type I interferon, and negative regulation of viral genome replication (Appendix, Figure 20A). The molecular function terms included double-stranded RNA binding, adenylyltransferase activity, ubiquitin-like protein ligase binding, and tumor necrosis factor receptor binding (Appendix, Figure 20B), whereas the cellular component terms included ribosomes and intracellular membrane-bound organelles (Appendix, Figure 20C).

Visualization of cancer hallmark enrichment

**Figure 16:**
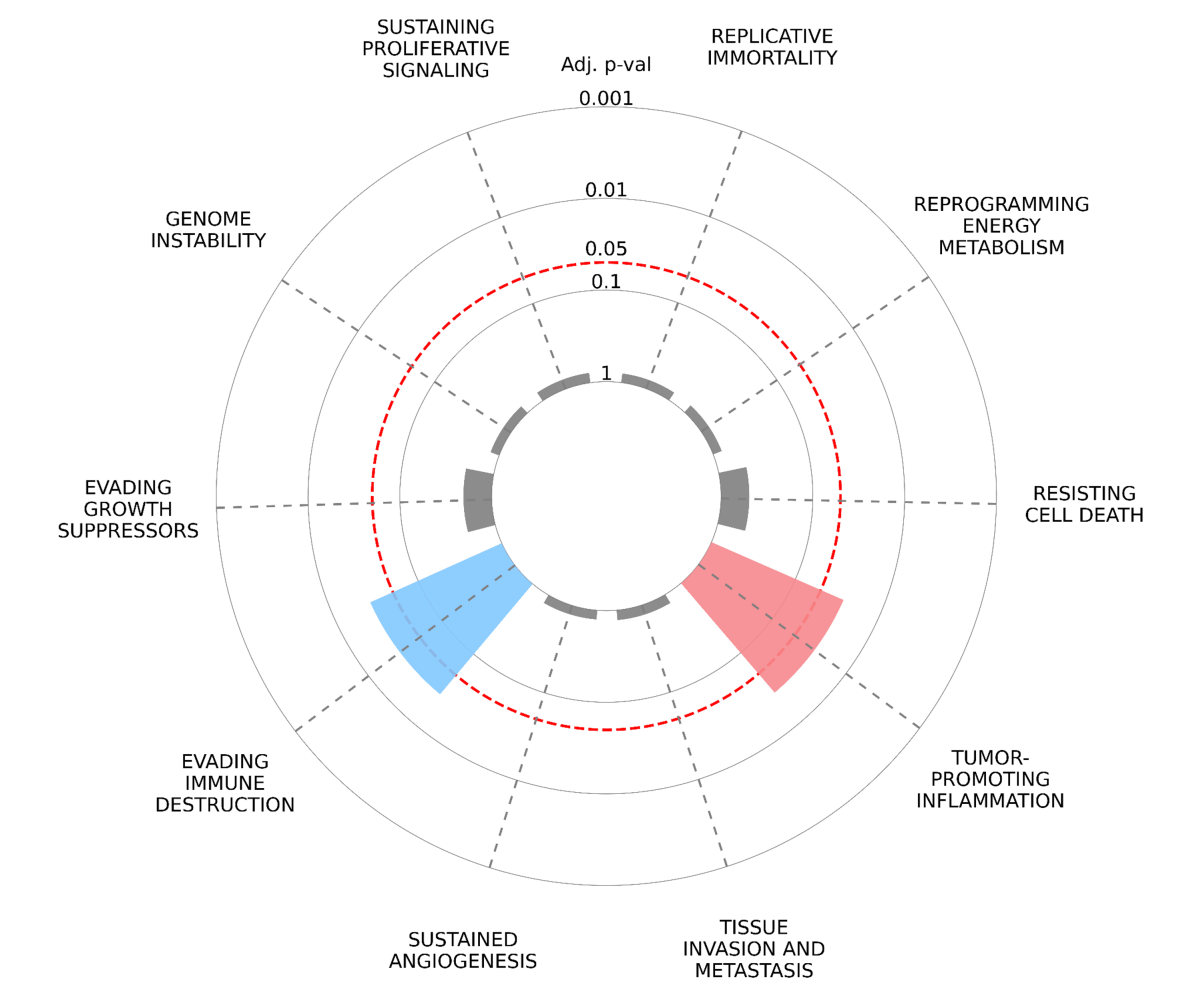
Tumor hallmarks associated with hub genes were identified using Cytoscape. Association between the main tumor hallmarks and the top 10 STAT1-related genes. STAT1: Signal transducer and activator of transcription 1

We found that three genes (STAT1, IRF7, and STAT2) were significantly involved in evading immune destruction and tumor-promoting inflammation (Figure 16).

## Discussion

Pan-cancer analysis has been extensively employed to identify potential biomarkers and reliable therapeutic targets [33,34]. In the current study, we investigated the expression pattern of STAT1 in physiological states by examining its expression in various normal tissues. We found that STAT1 expression was high in white blood cells, smooth muscle, retina, and skin (Figure 2). Upon studying the expression of STAT1 in different tumors, we found that STAT1 expression was significantly upregulated in 21 types of cancer. The significant cancers were BRCA, CESC, CHOL, COAD, DLBC, ESCA, GBM, HNSC, LGG, LIHC, LUAD, LUSC, OV, PAAD, READ, SKCM, STAD, TGCT, THCA, THYM, and UCEC (Figure 3).

Subsequently, the protein expression level of STAT1 was verified using the HPA database. Most cancers showed high-to-moderate immunoreactivity (Figure 4). In agreement with our results, a comprehensive analysis of 33 tumor types from the TCGA revealed that STAT1 is often upregulated in various cancers, including BRCA [1,7,35] and LUAD [36,37].

The UALCAN database revealed that the expression levels of STAT1 varied among different stages of different tumor types, including BRCA, CESC, ESCA, HNSC, LUAD, STAD, and UCEC. While there is no significant difference between normal tissue and tumor stages in PAAD and SKCM (Figure 5). Consistent with our results, previous studies on BRCA have demonstrated that the expression levels of STAT1 differ among the different clinical stages [38]. The upregulation of STAT1 in most cancers indicates its potential role in tumor progression, while the lack of significant differences in PAAD and SKCM highlights the complexity of its function in cancer biology.

**Figure 17:**
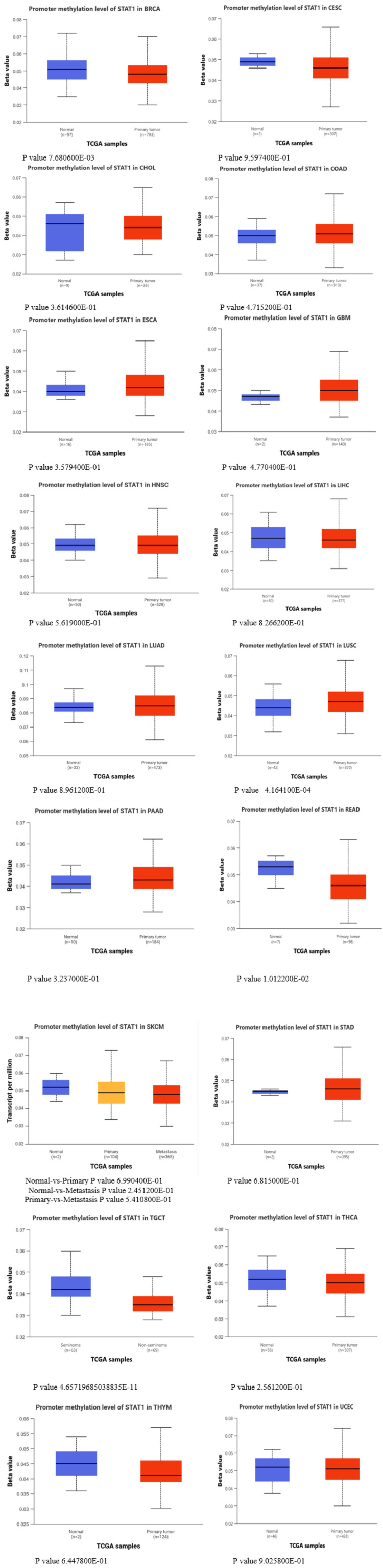
Promoter DNA methylation of the STAT1 comparison between normal and tumor samples using the UALCAN database. STAT1: Signal transducer and activator of transcription 1; UALCAN: University of Alabama at Birmingham Cancer Data Analysis Portal; BRCA: breast invasive carcinoma; CESC: cervical squamous cell carcinoma; ESCA: esophageal carcinoma; HNSC: head and neck squamous cell carcinoma; LUAD: lung adenocarcinoma; STAD: stomach adenocarcinoma; UCEC: uterine corpus endometrial carcinoma; LGG: low-grade gliomas; LIHC: liver hepatocellular carcinoma; PAAD: pancreatic adenocarcinoma; READ: rectum adenocarcinoma; OV: ovarian cancer; KICH: kidney chromophobe; TGCT: testicular germ cell tumor; THCA: thyroid cancer

**Figure 18:**
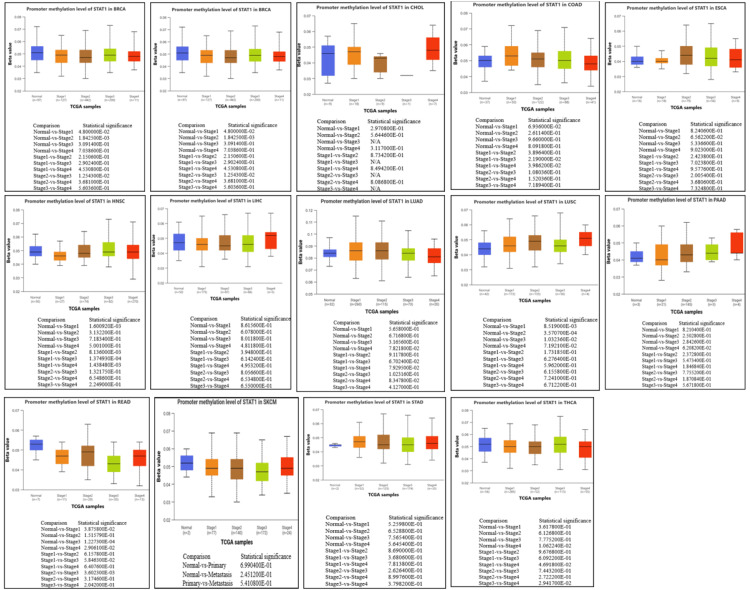
Promoter DNA methylation of STAT1 in normal and tumor samples based on individual cancer stages. *: p-value < 0.05; **: p-value <0.01; ***: p-value <0.001 STAT1: Signal transducer and activator of transcription 1; UALCAN: University of Alabama at Birmingham Cancer Data Analysis Portal; BRCA: breast invasive carcinoma; CESC: cervical squamous cell carcinoma; ESCA: esophageal carcinoma; HNSC: head and neck squamous cell carcinoma; LUAD: lung adenocarcinoma; STAD: stomach adenocarcinoma; UCEC: uterine corpus endometrial carcinoma; LGG: low-grade gliomas; LIHC: liver hepatocellular carcinoma; PAAD: pancreatic adenocarcinoma; READ: rectum adenocarcinoma; OV: ovarian cancer; KICH: kidney chromophobe; TGCT: testicular germ cell tumor; THCA: thyroid cancer

**Figure 19:**
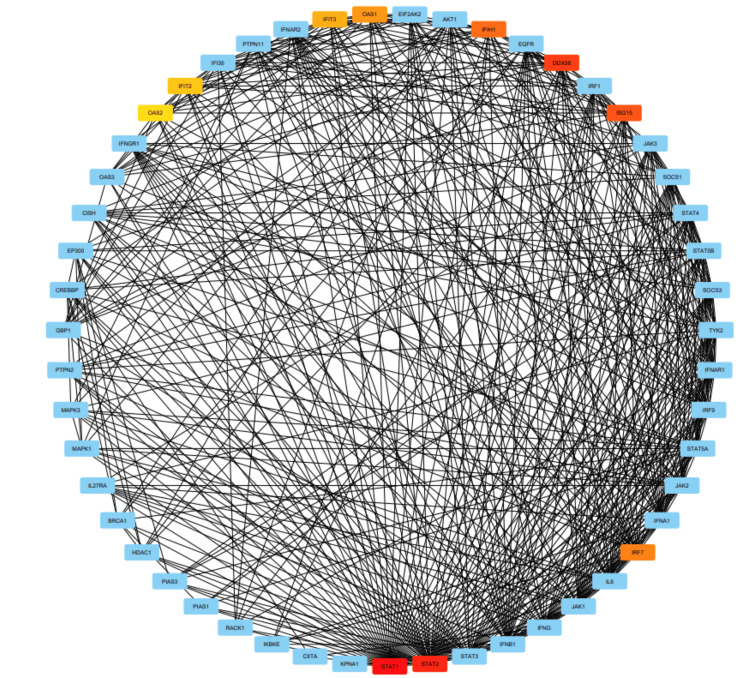
The protein protein interaction network of STAT1 using Cytoscape software. STAT1: Signal transducer and activator of transcription 1

DNA methylation, a complex epigenetic process, plays a vital role in controlling gene expression in both healthy and cancerous cells. This chemical modification can either decrease gene expression when it occurs at gene promoters (CpG methylation) or increase gene expression when it occurs within the gene [39]. In tumor cells, abnormal DNA methylation predominantly affects CpG islands within the regulatory elements of gene expression [40,41]. We studied the promoter methylation in all tumors with high STAT1 expression (Appendix, Figure 17). Our results revealed that the methylation levels of the STAT1 promoter in BRCA, READ, and TGCT were significantly (p < 0.05) lower than those in normal tissues, and there was a negative correlation between STAT1 promoter methylation levels and STAT1 expression. In LUSC, the promoter methylation was significantly higher in the tumor in comparison to normal tissue (Figure 6).

This suggests that STAT1 promoter methylation may play a role in regulating the expression of the STAT1 gene and explains the high STAT1 expression in these cancers.

Interestingly, promoter methylation analysis showed insignificant results in other tumors despite increased STAT1 expression. While hypomethylation of promoter regions is generally associated with increased gene expression, this relationship is not always straightforward. This apparent inconsistency may reflect complex regulatory mechanisms. The effect of hypomethylation on gene expression can vary depending on the cellular context, including the presence of transcription factors, chromatin structure, and other epigenetic modifications. For instance, in some cases, hypomethylation may not lead to increased expression if other regulatory mechanisms are at play that inhibit transcription [42,43].

CancerSEA was used to analyze the correlation between STAT1 and multiple functional states of cancer cells at the single-cell level. We found that the upregulated expression of STAT1 was both positively and negatively correlated with angiogenesis, apoptosis, cell cycle, differentiation, DNA damage, DNA repair, epithelial-mesenchymal transition (EMT), inflammation, invasion, metastasis, and stemness. Negative correlations were observed between STAT1 expression, hypoxia, and cell proliferation (Figure 8). STAT1 influences many functional states of cancer cells and their interactions within the tumor microenvironment (TME) [44]. Our findings indicate that STAT1 plays multifaceted roles in cancer, demonstrating both positive and negative correlations with various cellular processes.

STAT1 is a known negative regulator of angiogenesis [45]. In agreement with our results, studies have shown that activation of STAT1 in endothelial cells through the action of interferon-gamma (IFN-γ) leads to the inhibition of endothelial cell growth mediated by the suppression of pro-angiogenic factors, such as vascular endothelial growth factor (VEGF) and angiopoietin-2 [45,46]. Additionally, STAT1 promotes the expression of inducible nitric oxide (NO) synthase (iNOS), which is linked to increased NO production and enhanced angiogenesis [47]. The induction of NO may also initiate cellular apoptosis by augmenting p53 expression [48]. Moreover, STAT1 is implicated in the regulation of angiogenic factors, as in LUAD, in which IL-17 promotes the production of angiogenic factors (IL-6 and VEGF) through STAT1 signaling, thereby supporting angiogenesis [49].

STAT1 is primarily known for its role in mediating apoptosis by upregulating pro-apoptotic genes, including those encoding caspases, FAS, and TRAIL [50,51]. However, under certain conditions, STAT1 inhibits apoptosis, thereby contributing to cell survival. This dual functionality is context-dependent and influenced by various signaling pathways and cellular environments [50]. Conversely, STAT1 exerts anti-apoptotic effects. It promotes the expression of anti-apoptotic proteins, Mcl-1 [52], and PD-L1 [53].

Furthermore, we studied the correlation between STAT1 expression and OS in various tumor types. We found that a high expression level of STAT1 was associated with better OS in HNSC, OV, READ, SKCM, and THCA. This suggests that STAT1 has a better prognostic predictive value in these cancers. However, higher STAT1 expression in LGG, LUAD, PAAD, THYM, and UCEC was associated with worse OS (Figures 9-10). Our survival results suggest that STAT1 expression exhibits different effects on patient survival among tumors, indicating a different role played by STAT1 in the biological characteristics of various tumors. In agreement with our results, previous studies have found that a high level of STAT1 is associated with better OS in OV [47], colorectal cancer [54,55], and SKCM [56,57]. High expression levels of STAT1 have been correlated with poor OS in certain cancers, including LGG, LUAD, and PAAD [56].

We studied four types of immune cell infiltration, and the expression of STAT1 was found to be significantly positively correlated with the infiltration of B cells, CD8+ cells, CD4+ cells, and macrophages in LGG, LUAD, PAAD, THYM, and UCEC. Overall, the expression of STAT1 was significantly positively correlated with the infiltration of B cells, CD8+ cells, CD4+ cells, and macrophages in LGG, LUAD, and PAAD. Moreover, we found that the expression of STAT1 was significantly correlated with B, CD8+, and CD4+ cells in UCEC. Additionally, we found that the expression of STAT1 was significantly negatively correlated with the infiltration of CD8+, CD4+, and macrophages in the THYM (Figure 11). These findings suggest that the carcinogenic mechanism of STAT1 may be related to antitumor immunity and that STAT1 may potentially impact immunotherapy.

We used the TISIDB database to analyze STAT1 expression in the immune subtypes of tumors with upregulated STAT1 expression, associated with worse prognosis. The results showed that STAT1 expression differed significantly in LGG (four subtypes), LUAD (five subtypes), PAAD (five subtypes), and UCEC (five subtypes) (Figure 12). STAT1 is abnormally expressed, leading to outcomes that may not be representative of the overall cancer population. This variation can help explain why changes in STAT1 expression do not influence survival rates in some cancers, whereas its varying expression across different immune subtypes can significantly impact the prognosis of different cancer types.

**Figure 20:**
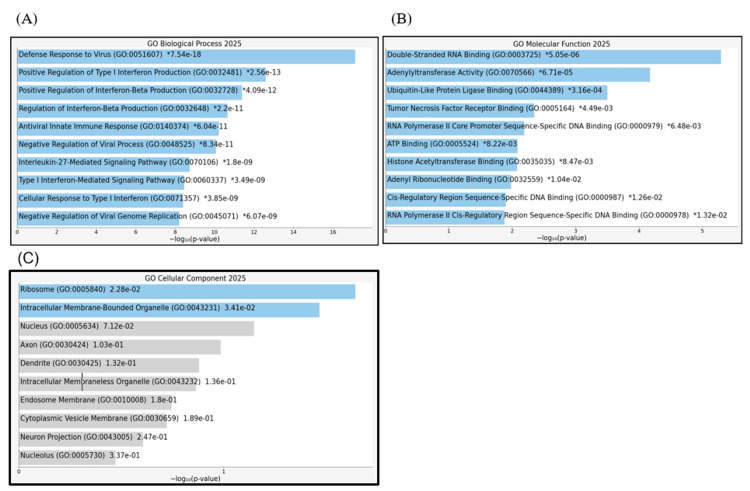
Gene ontology of the 10 hub genes using the Appyters feature of Enrichr. (A) GO-biological process. (B) GO-molecular function. (C) GO-cellular component

We used STRING to study PPIs. The top 50 proteins interacting with STAT1 are shown in Figure 13. KEGG enrichment analysis was used to explore genes co-expressed with STAT1 and related biological pathways. The analysis showed that STAT1-related genes and proteins mainly acted on the JAK-STAT signaling pathway, viral infections (influenza A, measles, hepatitis C, herpes simplex virus 1, hepatitis B, and Epstein-Barr virus), pathways in cancer, Kaposi sarcoma-associated herpesvirus infection, and human papillomavirus infection (Figure 14). Additionally, we found 22 proteins, including STAT1, that were significantly enriched in pathways related to cancer, as highlighted in red in Figure 13. Moreover, we constructed a PPI network using Cytoscape (Appendix, Figure 20) and identified 10 hub genes using the cytoHubba plugin. The hub genes were STAT1, STAT2, ISG15, IFIT2, OAS1, IRF7, IFIT3, IFIH1, DDX58, and OAS2 (Figure 15).

The "Hallmarks of Cancer" framework offers fundamental organizing principles that are shared across diverse cancers. Recognizing these hallmarks is valuable for cancer prevention, diagnosis, and the development of novel therapeutic agents. We found that three genes (STAT1, IRF7, and STAT2) out of the 10 hub genes were involved in both evading immune destruction (p = 0.008306) and tumor-promoting inflammation (p = 0.008935), as presented in Figure 16.

## Conclusions

In summary, our pan-cancer analysis of STAT1 showed a statistically significant association between gene expression and DNA methylation, functional states, clinical prognosis, tumor immune infiltration, and immune subtypes in multiple tumors. We also studied STAT1-related protein interactions, KEGG pathways, and the cancer hallmarks of the hub genes.

Our study is limited by the lack of experimental validation. Additionally, it is a bioinformatics analysis based on publicly available databases with a small sample size. Increasing the sample size may enhance the generalizability of the results. Nevertheless, to the best of our knowledge, this study represents a comprehensive investigation of the relationship between STAT1 methylation across various tumors, the correlation between STAT1 expression and functional states in cancer, STAT1-related protein interactions, gene enrichment analysis, and hallmarks of cancer analysis. These findings can help further clarify the role of STAT1 in tumorigenesis and development and provide a new reference for potential applications in immunotherapy. Despite the recognition of STAT1's involvement in various signaling pathways, the precise molecular mechanisms by which STAT1 influences tumor behavior remain poorly understood. However, the specific pathways and interactions that lead to its tumor-promoting or suppressive effects have not been fully elucidated. Further research is needed to dissect these mechanisms, particularly in the context of immune evasion and therapeutic resistance.
